# High preoperative plasma vasohibin‐1 concentration predicts better prognosis in patients with non–small cell lung carcinoma

**DOI:** 10.1002/hsr2.40

**Published:** 2018-05-17

**Authors:** Tatsuaki Watanabe, Tomoko Hosaka, Kaori Ohmori‐Matsuda, Yasuhiro Suzuki, Hirotoshi Suzuki, Hiroshi Yabuki, Yasushi Matsuda, Masafumi Noda, Akira Sakurada, Yoshinori Okada, Yasufumi Sato

**Affiliations:** ^1^ Department of Thoracic Surgery, Institute of Development, Aging and Cancer Tohoku University Sendai Japan; ^2^ Department of Vascular Biology, Institute of Development, Aging and Cancer Tohoku University Sendai Japan; ^3^ Department of Thoracic Surgery Japan Organization of Occupational Health and Safety Tohoku Rosai Hospital Sendai Japan; ^4^ Division of Epidemiology, Department of Public Health and Forensic Medicine Tohoku University Graduate School of Medicine Sendai Japan

**Keywords:** biomarker, non–small cell lung cancer, plasma concentration, vasohibin‐1

## Abstract

**Background and Aim:**

Vasohibin‐1 (VASH1) is an angiogenesis inhibitor synthesized and secreted by endothelial cells, whose expression is induced by angiogenic stimuli such as vascular endothelial growth factor. We have previously demonstrated that VASH1 is immunohistochemically evident in endothelial cells in the tumor microenvironment of patients with non–small cell lung cancer (NSCLC) and is positively correlated with that of vascular endothelial growth factor in cancer cells. Here, we determined the preoperative plasma concentration of VASH1 in patients with NSCLC and evaluated the association between the preoperative VASH1 levels and certain outcomes.

**Methods:**

We analyzed presurgical plasma VASH1 concentrations in a total of 79 lung cancer patients (51 males and 28 females; 34‐83 y of age; 46 adenocarcinomas, 27 squamous cell carcinomas, and 6 other types) who underwent lung resection. The impact of preoperative VASH1 level was analyzed using clinical characteristics and prognosis.

**Results:**

Plasma VASH1 concentration ranged from 34.1 to 1190.4 fmol/mL. We divided the patients into 3 groups according to plasma VASH1 level for this assessment: low VASH1 group (n = 26), medium VASH1 group (n = 27), and high VASH1 group (n = 26). The death and recurrence rates of the high, medium, and low VASH1 groups were 5.5, 16.2, and 12.7 per 100 person‐years, respectively. Multivariate adjusted hazard ratio of death and recurrence of the high VASH1 group was lower than that of the low VASH1 group (hazard ratio 0.42; 95% CI 0.17‐0.99).

**Conclusion:**

The present analysis suggests that high preoperative plasma VASH1 concentration is associated with better prognosis in patients with NSCLC. We propose preoperative VASH1 level as a biomarker for the prognosis of patients with non–small cell lung carcinoma.

## 1 INTRODUCTION

The leading cause of cancer‐related deaths in the world is lung cancer.^1^ Non–small cell lung cancer (NSCLC) represents the majority of lung cancer cases, and up to 20% of the total cancer‐related deaths is derived from NSCLC.^2^ Surgical treatment is a standard treatment for early‐stage NSCLC.^3^ The outcome of surgical treatment has been improved due to better operation procedures, optimal postoperative care, and advances in chemotherapy.^4^, ^5^ However, the 5‐year survival rate of NSCLC remains only around 50%.^5^

Solid tumor is composed of tumor cells, tumor vessels, infiltrated stromal and inflammatory cells, and accumulated extracellular matrices.^6^ Tumor vessels lined with endothelial cells (ECs) are critical for tumor progression, as they supply oxygen and nutrients to the cancer cells and also act as a doorway for cancer cells for metastatic dissemination. Tumor vessels are formed by a process known as angiogenesis or neovascularization,^7^ and this process is controlled by a local balance between its stimulators and inhibitors.^8^

Our analysis has identified vasohibin‐1 (VASH1) as an endogenous angiogenesis inhibitor synthesized and secreted by ECs, whose expression is induced by representative angiogenesis stimulators such as vascular endothelial growth factor (VEGF) and fibroblast growth factor 2.^9^ Thus, we proposed VASH1 as a negative feedback regulator of angiogenesis.^9^ Indeed, our subsequent analyses have revealed that VASH1 is immunohistochemically detectable in ECs of tumor vessels, but not in ECs of normal vessels in human,^10^ and inhibits tumor growth and metastasis in experimental murine models.^10^, ^11^, ^12^ Due to the characteristics of VASH1 as an angiogenesis inhibitor, increased expression of VASH1 is expected to be beneficial and should contribute to a better prognosis of cancers. However, contrary to this expectation, immunohistochemical analyses of pathological human sections have revealed that increased VASH1 immunostaining in ECs of tumor vessels is predictive of poor clinical outcome in multiple cancer types including NSCLC^13^, ^14^, ^15^, ^16^, ^17^, ^18^, ^19^, ^20^; an exception to this is renal cell carcinoma.^21^

The mature VASH1 protein is composed of 365 amino acids and has a predicted molecular weight of 44 kDa. This protein is posttranslationally degraded after its secretion into at least 2 truncated forms: a 36‐kDa protein and a 29‐kDa protein.^22^ The 36‐kDa VASH1 protein is an N‐terminally truncated form retaining antiangiogenic activity, whereas the 29‐kDa VASH1 protein is truncated at both the N‐terminal and C‐terminal ends and loses antiangiogenic activity.^22^ Immunohistochemical staining of VASH1 protein within ECs may thus not immediately correlate with antiangiogenic activity of VASH1 after secretion.

We have developed a VASH1 enzyme‐linked immunosorbent assay (ELISA) that can detect the 44‐ and 36‐kDa VASH1 forms that preserve antiangiogenic activity.^11^, ^22^ Here, we applied this system, determined the preoperative plasma VASH1 concentration in patients with lung cancer, and evaluated its potential value as a prognostic factor in patients after surgery.

## 2 MATERIALS AND METHODS

### 2.1 Patient enrollment and sample and data collection

A total of 147 patients with lung tumors underwent operation at Tohoku University Hospital, Japan, from April 2005 to January 2007. Among them, 99 patients who consented to this study were subject to plasma VASH1 level quantitation before surgery. Twenty patients were excluded for the following reasons: benign disease (6), metastatic diseases (2), small cell lung cancer (1), stage IV (7), and insufficient data (4).

A total of 79 patients were followed up by experienced clinicians with standard physical examination, including chest X‐rays, chest computed tomography scans, and tumor marker assessments, according to histological types for at least 5 years after surgery, unless recurrences were detected or the patients dropped out from the follow‐up. Data were collected through in‐person interviews using a standard epidemiological questionnaire, including age, sex, smoking status, performance status, and permanent residence. Clinical information including pathological stage, sex, smoking status, performance status, and prognosis were assessed using medical records. This staging was based on *Classification of Lung Cancer*, Seventh Edition, by The Japan Lung Cancer Society.^22^ Date of death and causes of death were obtained by mail questionnaires or permission from the Ministry of Justice after the follow‐up. This study was approved by the ethical review board of the Tohoku University Hospital and was conducted in accordance with the principles specified in the Declaration of Helsinki.

### 2.2 Enzyme‐linked immunosorbent assay for VASH1

Enzyme‐linked immunosorbent assay for VASH1 was performed as described previously.^11^, ^23^ A peptide corresponding to Gly286‐Arg299 (VC) and Ala217‐Lys229 (VR1) of human VASH1 protein (Q7L8A9) were conjugated with keyhole limpet hemocyanin, and these antigens were immunized to A/J mice, and several monoclonal antibodies (mAbs) were prepared.^9^ Among them, we found that a particular combination of VC‐derived clones, recognizing Gly286‐Arg299, and VR1‐derived clones, recognizing Ala217‐Lys229, was the best for the VASH1 ELISA.^11^ We used VC1‐derived clone 12F6 and VR‐derived clone 12E7 for plate coating and horseradish peroxidase (HRP) labeling, respectively. A 100‐μL aliquot of anti‐VASH1 mAb VR1 (10 μg/mL) in phosphate‐buffered saline (PBS) was added to each well of an immunoplate and incubated overnight at 4°C. Each well was washed with saline and blocked with 200 μL of PBS containing 0.5% bovine serum albumin. An aliquot of the sample (25 μL) or standard diluted with assay buffer (100mM PBS, pH 7.0, containing 0.5% bovine serum albumin) and 75‐μL assay buffer were added to each well. The plate was left at 4°C overnight and then washed 3 times with washing buffer. Next, 100 μL of antivasohibin mAb‐HRP (1 μg/mL VC‐HRP in assay buffer) was added to each well, and the samples were incubated at room temperature for 2 hours. The plate was washed 3 times with washing buffer, and the immunoreactivity was visualized by adding 100 μL of substrate solution (Color‐Burst Blue, AlerCHECH, Springvale, Maine), leaving the plate to stand for 30 minutes at room temperature. The reaction was stopped by addition of stop buffer, and the absorbance at 450 nm was measured using ARVO HTS (PerkinElmer Life Science, Yokohama). This ELISA system detects the 44‐ and 36‐kDa VASH1 form, but not the 29‐kDa VASH1 form.^23^

### 2.3 Statistical analysis

The association between serum VASH1 levels and baseline characteristics was evaluated by χ^2^ test, *t* test, or one‐way analysis of variance, as appropriate. To compare the relapse‐free survival (RFS), we used Kaplan‐Meier survival plots and log‐rank statistics. Multivariate analyses were performed using the Cox proportional hazards regression analysis. We counted the number of person‐years of follow‐up for each patient from the operation day until the date of death, the relapse of the lung cancer, or the last confirmation of existence, whichever occurred first.

All statistical analyses were performed using SAS V.9.3 (SAS Institute, Cary, North Carolina, USA) and GraphPad Prism 6 (GraphPad Software, San Diego, CA), and all tests were 2‐sided, with .05 defined as the level of significance.

## 3 RESULTS

### 3.1 Plasma VASH1 level stratified according to sex, histology, and stages

Clinical characteristics of 79 patients are summarized in Table 1. Among 79 patients, 51 were male and 28 were female. The age ranged from 34 to 83 (median, 67). The numbers of patients according to pathological stages were as follows: IA 31, IB 20, IIA 13, IIB 6, and IIIA 9. The numbers for each pathological type were as follows: 45 adenocarcinomas, 28 squamous cell carcinomas, and 6 others (2 large cell neuroendocrine carcinoma, 1 carcinoid, 1 combination of squamous cell carcinoma and adenocarcinoma, 1 large cell lung carcinoma, and 1 adenosquamous carcinoma).

**Table 1:** Characteristics of the patients and the 3 groups^a^

Parameter	Patients, n (%)	Plasma VASH1 Level, fmol/mL	*P* Value	Low VASH1	Medium VASH1	High VASH1	*P* Value
Age, y							.19^b^
Median	67			69	68	64	
Range	34‐83			52‐81	51‐77	34‐83	
Age ≧ 75 y	15 (19.0%)						
Gender			.34^c^				.92^d^
Male	51 (64.6%)	274.7 ± 155.3 (74.2‐1190.4)		17	18	16	
Female	28 (35.4%)	315.2 ± 213.4 (34.1‐1039.0)		9	9	10	
Smoking history							.97^e^
Never smoker	22 (27.8%)	310.0 ± 217.2 (34.1‐1039.0)	.52^c^	7	8	7	
Ex‐smoker	57 (72.2%)	280.9 ± 161.5 (74.2‐1190.4)		19	19	19	
Brinkman index range (median)	0‐3120 (600)			0‐1625 (600)	0‐3120 (800)	0‐2400 (500)	.73^c^
Performance status							
0	73 (92.4%)	290.6 ± 183.7 (34.1‐1190.4)	.79^c^	25	23	25	
1	6 (7.6%)	270.6 ± 80.0 (180.3‐418.2)		1	4	1	
Pathological stage			.99^b^				
Stage IA	31 (38.8%)	294.6 ± 147.4 (118.7‐766.7)		9	9	14	.24^f^
Stage IB	20 (26.3%)	297.7 ± 242.9 (34.1‐1190.4)		9	3	8	
Stage IIA	13 (16.3%)	290.9 ± 230.2 (142.0‐1039.0)		6	5	2	
Stage IIB	6 (7.5%)	271.3 ± 84.2 (155.2‐418.2)		1	4	1	
Stage IIIA	9 (11.3%)	259.8 ± 31.8 (193.1‐301.3)		1	6	2	
Pathology			.67^d^				.82^e^
Adenocarcinoma	45 (57.0)	282.9 ± 180.2 (34.1‐1039.0)		16	14	15	
Squamous cell carcinoma	28 (35.4%)	302.0 ± 190.7 (141.6‐1190.4)		8	10	10	
Others	6 (7.6%)						

Abbreviation: VASH1, vasohibin‐1.

a Low VASH1, approximately 222; medium VASH1, 223‐281; and high VASH1, >282 fmol/mL.

b One‐way analysis of variance.

c *t* test.

d *t* test for adenocarcinoma vs squamous carcinoma.

e χ^2^ test.

f χ^2^ test for IA vs non‐IA.

**Figure 1:**
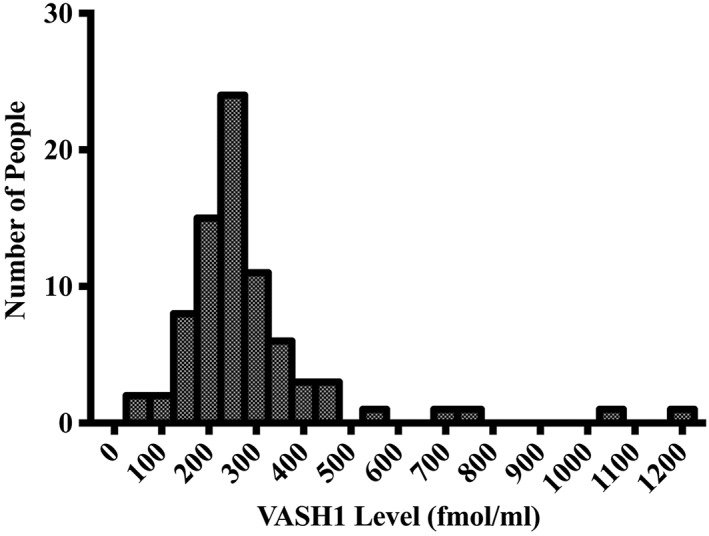
Histogram of plasma VASH1 levels of the 79 patients. The plasma VASH1 levels of 4 patients were over 2 SD. VASH1 indicates vasohibin‐1

The plasma VASH1 levels ranged from 34.1 to 1190.4 fmol/mL (289.4 ± 177.7 fmol/mL). The plasma VASH1 levels of 4 patients were over 2 SD higher (Figure 1). The clinical features of these 4 patients were stages IA, IB, IIA, and IIA, respectively; recurrence was observed in 2 of these patients, and 2 were ex‐smokers. Three of these patients were female.

The plasma VASH1 level of male patients was 274.7 ± 155.3 fmol/mL and that of female patients was 310.0 ± 217.2 fmol/mL. There was no significant difference in plasma VASH1 level between males and females. The plasma VASH1 levels of patients according to lung cancer stages were as follows: IA 294.6 ± 147.4 fmol/mL; IB 297.7 ± 242.9 fmol/mL; IIA 290.9 ± 230.2 fmol/mL; IIB 271.3 ± 84.2 fmol/mL; and IIIA 259.8 ± 31.8 fmol/mL. There were no significant differences in VASH1 levels among lung cancer stages.

The plasma VASH1 levels of patients with adenocarcinoma were 282.9 ± 180.2 fmol/mL, and those of patients with squamous cell carcinoma were 302.0 ± 190.7 fmol/mL. There was no significant difference in plasma VASH1 level between adenocarcinoma and squamous cell carcinoma.

### 3.2 Comparing plasma VASH1 level in relapse‐free survival

We divided patients into 3 tertiles, according to their plasma VASH1 levels, for the assessment of RFS: low VASH1 group (n = 26, <222 fmol/mL), medium VASH1 group (n = 27, 223‐281 fmol/mL), and high VASH1 group (n = 26, >282 fmol/mL). There were no significant differences on the basis of sex, smoking history, stage, and histology, among the 3 groups (Table 1). The follow‐up period after surgery ranged from 146 to 3052 days for all patients, and from 434 to 3052 days for survivors.

**Figure 2:**
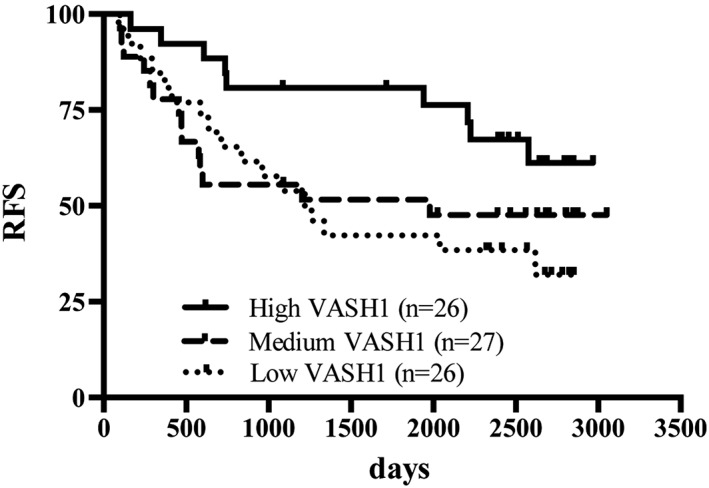
Relapse‐free survival of high, medium, and low groups. Relapse‐free survival rates were 61.2% in high VASH1 group, 47.6% in medium VASH1 group, and 32.1% in low VASH1 group. High VASH1 group had better prognosis than had low VASH1 level patients. *P* = .056, log‐rank test. VASH1 indicates vasohibin‐1

**Figure 3:**
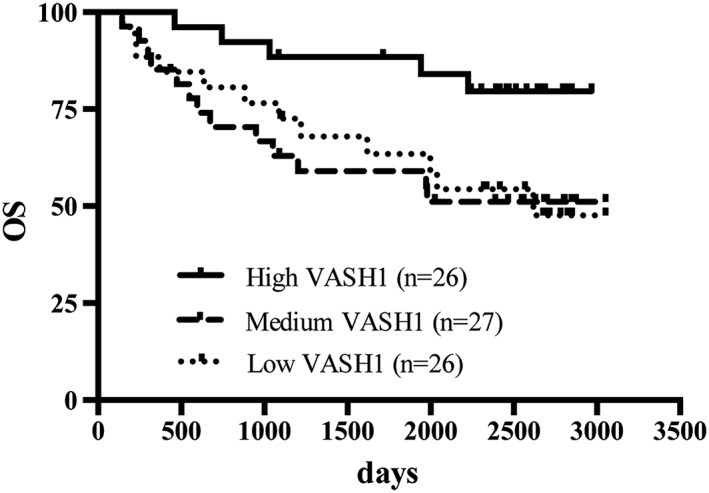
OS of high, medium, and low groups. OS rates were 79.6% in high VASH1 group, 51.2% in medium VASH1 group, and 47.6% in low VASH1 group. High VASH1 group had better prognosis than had low VASH1 level patients. *P* = .054, log‐rank test. OS indicates overall survival; VASH1, vasohibin‐1

Factors affecting relapse or survival were assessed by Cox proportional hazards regression analysis and Kaplan‐Meier estimates. The RFS rate of the high, medium, and low VASH1 groups were 61.2%, 47.6%, and 32.1%, respectively (Figure 2). The death and recurrence rate of the high, medium, and low VASH1 groups were 5.5, 16.2, and 12.7 per 100 person‐years, respectively. The high VASH1 group showed better prognosis than did the low VASH1 group (crude hazard ratio [HR] 0.38; 95% CI 0.16‐0.87). A multivariate Cox proportional hazards model was performed (Table 2). Factors such as age, sex, stage, and baseline performance status were considered. The results still demonstrated an independent favorable effect of plasma VASH1 levels for the prognosis of lung cancer patients (HR 0.42; 95% CI 0.17‐0.99). The survival curves of the low VASH1 group and medium VASH1 group intersected at 3 years after surgery. As such, we analyzed the data before 3 years after surgery and after 3 years after surgery. The effects of VASH1 levels may differ before 3 years after surgery and after 3 years after surgery. Overall survival showed similar trend as RFS (Figure 3 and Table 3).

**Table 2:** Hazard ratio (HR) and 95% CI of death and lung cancer death and recurrence^a^

Analysis by the multivariate Cox proportional hazards model	Low VASH1	Medium VASH1	High VASH1
All Periods			
Person‐years	104.9	110.7	144.3
Death and recurrence rate (per 100 person‐years)	16.2	12.7	5.5
Crude HR (95% CI)	Ref	0.84 (0.41‐1.71)	0.38 (0.16‐0.87)
Multivariate adjusted HR (95% CI)	Ref	0.83 (0.37‐1.88)	0.42 (0.17‐0.99)
First 3 y After the Operation			
Person‐years	59.4	56.8	70.1
Death and recurrence rate (per 100 person‐years)	20.2	21.1	7.1
Crude HR (95% CI)	Ref	1.06 (0.48‐2.36)	0.36 (0.13‐1.01)
Multivariate adjusted HR (95% CI)	Ref	1.16 (0.46‐2.96)	0.40 (0.14‐1.17)
More Than 3 y After the Operation			
Person‐years	45.5	53.9	74.2
Death and recurrence rate (per 100 person‐years)	11.0	3.7	4.0
Crude HR (95% CI)	Ref	0.36 (0.070‐1.88)	0.39 (0.093‐1.63)
Multivariate adjusted HR (95% CI)	Ref	0.20 (0.026‐1.60)	0.28 (0.052‐1.49)

Abbreviation: VASH1, vasohibin‐1.

a Multivariate HR (95% CI) has been adjusted for age, sex, baseline performance status (0, 1, or more), and pathological stage (stages IA, IB, and IIA/IIB/IIIA).

**Table 3:** Hazard ratio (HR) and 95% CI of death^a^

Analysis before 3 years post‐surgery	Low VASH1	Medium VASH1	High VASH1
All Periods			
Person‐years	123.0	125.7	157.1
Death and recurrence rate (per 100 person‐years)	9.8	10.3	3.2
Crude HR (95% CI)	Ref	1.08 (0.49‐2.36)	0.34 (0.12‐0.96)
Multivariate adjusted HR (95% CI)	Ref	1.11 (0.49‐2.52)	0.37 (0.13‐1.08)
First 3 y After the Operation			
Person‐years	65.0	65.5	75.1
Death and recurrence rate (per 100 person‐years)	10.8	15.3	4.0
Crude HR (95% CI)	Ref	1.41 (0.54‐3.71)	0.37 (0.096‐1.43)
Multivariate adjusted HR (95% CI)	Ref	1.34 (0.44‐4.07)	0.43 (0.11‐1.73)
More Than 3 y After the Operation			
Person‐years	58.0	60.2	82.0
Death and recurrence rate (per 100 person‐years)	8.6	5.0	2.4
Crude HR (95% CI)	Ref	0.61 (0.14‐2.53)	0.29 (0.056‐1.49)
Multivariate adjusted HR (95% CI)	Ref	0.54 (0.095‐3.03)	0.27 (0.045‐1.63)

Abbreviation: VASH1, vasohibin‐1.

a Multivariate HR (95% CI) has been adjusted for age, sex, baseline performance status (0, 1, or more), and pathological stage (stages IA, IB, and IIA/IIB/IIIA).

The association between VASH1 and RFS was analyzed using VASH1 as a continuous value. We could not find a significant linear association between VASH1 values and RFS. Crude and adjusted HRs for 100‐fmol/mL increase in VASH1 were 0.89 (95% CI 0.70‐1.13) and 0.92 (95% CI 0.74‐1.15), respectively, for all the observation periods. We also calculated HRs for first 3 years and 3 years or more after operation separately, but none of them were statistically significant (crude HRs 0.95 [95% CI 0.75‐1.19] and 0.97 [95% CI 0.79‐1.19]). For further evaluation, we redefined the VASH1 categories as follows: 205 or less, 206‐258, 259‐300, and 300 fmol/mL or more (Table 4). Among those with VASH1 259 fmol/mL or more, their HRs were significantly lower than those with 205 fmol/mL or less.

**Table 4:** Hazard ratio (HR) and 95% CI of death and lung cancer death and recurrence: 4 groups model^a^

Analysis after 3 years post‐surgery	Plasma VASH1, fmol/mL			
	Approximately 205	206‐258	259‐300	>301
All Periods				
Person‐years	65.4	96.4	88.3	109.7
Death and recurrence rate (per 100 person‐years)	21.4	9.3	12.5	4.6
Crude HR (95% CI)	Ref	0.48 (0.21‐1.11)	0.66 (0.30‐1.46)	0.24 (0.085‐0.66)
Multivariate adjusted HR (95% CI)	Ref	0.43 (0.18‐1.01)	0.71 (0.31‐1.64)	0.24 (0.081‐0.69)
First 3 y After the Operation				
Person‐years	38.4	50.1	43.1	54.6
Death and recurrence rate (per 100 person‐years)	28.6	12.0	18.6	7.3
Crude HR (95% CI)	Ref	0.43 (0.16‐1.17)	0.68 (0.27‐1.68)	0.26 (0.083‐0.82)
Multivariate adjusted HR (95% CI)	Ref	0.40 (0.15‐1.09)	0.79 (0.31‐2.02)	0.29 (0.087‐0.95)
More Than 3 y After the Operation				
Person‐years	26.9	46.3	45.2	55.1
Death and recurrence rate (per 100 person‐years)	11.1	6.5	6.6	1.8
Crude HR (95% CI)	Ref	0.61 (0.12‐3.03)	0.63 (0.13‐3.12)	0.17(0.018‐1.66)
Multivariate adjusted HR (95% CI)	Ref	0.49 (0.090‐2.65)	0.33 (0.046‐2.42)	0.099 (0.008‐1.20)

Abbreviation: VASH1, vasohibin‐1.

a Multivariate HR (95% CI) has been adjusted for age, sex, baseline performance status (0, 1, or more), and pathological stage (stages IA, IB, and IIA/IIB/IIIA).

## 4 DISCUSSION

The mature 44‐kDa VASH1 protein is degraded into a 36‐kDa protein and a 29‐kDa protein after secretion. The 36‐kDa protein retains antiangiogenic activity, whereas the 29‐kDa protein loses it.^11^ We have recently shown that this degradation is enhanced in the presence of cancer cells, which indicates that the VASH1 protein secreted by ECs is inactivated in the tumor microenvironment.^24^ Here, we applied a VASH1 ELISA assay, which detects antiangiogenic 44‐ and 36‐kDa protein, but not inactive 29‐kDa protein,^11^, ^23^ and evaluated the potential value of preoperative plasma VASH1 concentration as a prognostic factor in patients with resected lung cancer. Our analysis revealed that the high plasma VASH1 group showed a lower relapse and death rate than did the low VASH1 group throughout the observation period. We performed multiple comparisons and confirmed this result. This is the first demonstration that the determination of preoperative plasma VASH1 concentration can be used for prognostic assessment of lung cancer patients after surgery.

Multiple studies, including ours, have examined whether the immunohistochemical staining of VASH1 can be used as a biomarker in various cancer types. Contrary to expectations, most studies have shown that an increased intensity of VASH1 immunostaining in tumor vessels was associated with poor clinical outcomes.^13^, ^14^, ^15^, ^16^, ^17^, ^18^, ^19^, ^20^ Immunohistochemical analysis has shown the VASH1 protein in the cytoplasm of ECs, and the intensity of VASH1 immunostaining is positively correlated with that of VEGF in cancer cells.^10^, ^13^ As the degradation and inactivation of VASH1 after secretion are enhanced in the tumor microenvironment,^24^ immunohistochemical staining of VASH1 in ECs may not correlate with its antiangiogenic activity. Rather, it simply reflects the response of ECs to angiogenic stimulation by cancer cells. The source of plasma VASH1 is not determined at the moment. While it can be assumed to derive from tumor vasculature, it is also possible that VASH1 proteins in systemic circulation are derived from the normal vascular bed. Nevertheless, our present analysis is the first to propose the value of determining plasma VASH1 concentrations in lung cancer patients.

Blood sampling, or so‐called liquid biopsy, is now widely being tested for its practicality in early diagnosis, treatment monitoring, and/or prognosis assessment of cancer patients, including NSCLC.^25^ Such blood samples contain circulating tumor cells, circulating free DNA, exosomes, and tumor‐educated platelets, among other components.^25^ In particular, the value of circulating tumor cells in prognostic assessment of NSCLC is currently being investigated but is not yet applied in routine practice.^25^ We would like to suggest the implementation of determining plasma VASH1 concentrations as routine practice in regular clinics.

It has been shown that tail vein injection of adenovirus encoding human *VASH1* gene in experimental murine models increased plasma VASH1 concentration and inhibited tumor growth and metastasis.^10^, ^11^ The challenging question then is whether it would be possible to increase the plasma VASH1 concentration in medium and low VASH1 patients so as to improve their prognosis. Consequently, it is of critical importance to determine how to manipulate the plasma concentration of VASH1. We have recently demonstrated that persistent physical exercise raises plasma VASH1 concentrations in patients with peripheral vascular disease.^23^ Preoperative physical exercise has been reported to reduce the mortality of patients with NSCLC.^26^ In this context, it would be noteworthy to investigate whether preoperative physical exercise does indeed increase plasma VASH1 concentrations of patients with NSCLC, and if it is associated with an improvement in prognosis.

In summary, high preoperative plasma VASH1 concentration is associated with better prognosis in patients with lung cancer. We propose plasma VASH1 concentration as a suitable biomarker to assess the prognosis of patients with lung cancer after surgery.

## FUNDING

This work was supported by a Grant‐in‐Aid for Scientific Research on Priority Areas from the Japanese Ministry of Education, Science, Sports and Culture, and by Health (22112006).

## CONFLICT OF INTEREST

None.

## AUTHOR CONTRIBUTIONS

Conceptualization: Yasufumi Sato

Data curation: Tatsuaki Watanabe, Kaori Ohmori‐Matsuda

Formal analysis: Tatsuaki Watanabe

Investigation: Tomoko Hosaka

Project administration: Tomoko Hosaka, Yasuhiro Suzuki, Hirotoshi Suzuki, Hiroshi Yabuki, Yasushi Matsuda, Masafumi Noda, Akira Sakurada

Supervision: Yoshinori Okada

Writing – original draft preparation: Tatsuaki Watanabe, Tomoko Hosaka

Writing – review and editing: Yasufumi Sato
